# Alveolar Ridge Preservation With Fibro‐Gide or Connective Tissue Graft: A Randomized Controlled Trial of Soft and Hard Tissue Changes

**DOI:** 10.1002/cre2.929

**Published:** 2024-07-23

**Authors:** Ammar Ibrahim, Rowaida Saymeh

**Affiliations:** ^1^ Department of Periodontology, Faculty of Dental Medicine Damascus University Damascus Syria

**Keywords:** alveolar ridge preservation, extraction socket, soft tissue augmentation

## Abstract

**Objective:**

The aim of this study was to evaluate the effectiveness of a novel biomaterial (FG) for alveolar ridge preservation compared to CTG in terms of soft tissue thickness and bone dimensional changes.

**Materials and Methods:**

A randomized clinical trial was conducted on 30 patients who required extraction of 30 hopeless mandibular posterior teeth. All patients went through atraumatic tooth extraction, and then, they were randomly allocated to either a CTG, an FG, or a spontaneous healing (SH) group (1:1:1). All patients received a dental implant placed 6 months postoperatively. The soft tissue thickness and bone dimensional changes were measured before and 6 months after the procedure.

**Results:**

The study's analysis revealed statistically significant differences in buccal gingival thickness and dimensional bone changes across the three examined groups after 6 months (*p* < 0.05). The SH group had lower gingival thickness (1.31 ± 0.65 mm) and higher vertical resorption (−1.46 ± 1.67 mm at the buccal aspect) compared with the CTG and FG groups. The CTG and FG groups had similar gingival thickness (2.42 ± 0.70 and 3.00 ± 0.71 mm, respectively) and bone width reduction (+0.86 ± 2.31 and +0.93 ± 2.38 mm, respectively), whereas the CTG group had lower vertical bone loss (−0.30 ± 1.09 mm at the buccal aspect) than the FG group (−0.47 ± 2.30 mm at the buccal aspect).

**Conclusion:**

FG and CTG demonstrate equivalent soft tissue thickness and comparable horizontal bone dimension outcomes in ARP.

## Clinical Relevance

*Scientific rationale*: Tooth extraction can lead to alveolar ridge resorption, which may impair the aesthetic and functional outcomes of implant‐supported prostheses. Alveolar ridge preservation (ARP) is a surgical technique that aims to minimize postextraction bone dimensional changes. ARP has been shown to reduce the horizontal and vertical bone resorption, maintain the soft tissue contour, and facilitate the implant placement. However, ARP also has some drawbacks, such as the risk of infection, membrane exposure, graft resorption, and increased cost and complexity. Therefore, it is desirable to optimize the ARP protocol by modifying the graft or membrane materials or by using alternative approaches.

*Principal findings*: Fibro‐Gide (FG) and connective tissue graft can preserve alveolar ridge and buccal soft tissue dimensions after tooth extraction. They can reduce bone resorption and maintain soft tissue thickness compared to spontaneous healing.

*Practical implications*: Our study suggests that alveolar ridge preservation with FG is a feasible alternative to conventional ARP with connective tissue graft in extraction sockets.

## 1 Introduction

Tooth extraction is normally followed by the resorption of alveolar bone, which may result in aesthetic and biologic problems. According to the scientific literature, alveolar bone resorption in the vertical direction can reach 11%–22% 6 months after the tooth extraction, whereas the resorption in the horizontal direction can reach 29%–63% of the original bone width before tooth extraction (Esposito et al. 2009; Hämmerle, Araújo, and Simion 2012; Ten Heggeler, Slot, and Van der Weijden 2011; Wang and Lang 2012). The success of osseointegrated dental implants is dependent on the presence of sufficient bone volume at the site of implant placement (Meloni et al. 2015; Papaspyridakos et al. 2012).

Alveolar ridge preservation (ARP) can be defined as a surgical intervention that aims to reduce alveolar bone resorption after tooth extraction (Araújo and Lindhe 2009). ARP methodologies may encompass the utilization of a biomaterial, such as bone particles, collagen sponges, or autologous blood‐derived products, to fill the extraction socket, a process typically referred to as socket grafting (ARP‐SG). Alternatively, ARP may involve the exclusive use of a barrier material, either autogenous or exogenous, to safeguard the bone compartment beneath, a technique known as socket sealing. These interventions may also be combined, promoting healing by primary intention following flap advancement (covered) or by secondary intention (exposed) (Avila‐Ortiz, Chambrone, and Vignoletti 2019). Different ARP procedures have been developed, using different bone substitutes, such as xenografts (Sivolella et al. 2020), allografts (Nelson and Mealey 2020), alloplasts (Machtei et al. 2019), and xenografts embedded in collagen (Ackermann 2009). However, there is no specific guideline as yet on a specific material or technique being superior to use, and there is a need for further studies to clarify these issues (Meloni et al. 2015).

CTG is a key material used in the socket seal technique, which is an integral part of ARP strategies (MacBeth et al. 2017). Socket seal surgery can be defined as “a procedure that, through soft tissue grafts or biomaterials, can seal the socket, complementing the guided bone regeneration or acting alone to preserve the soft tissues, thereby preventing its collapse” (López‐Pacheco et al. 2021). This technique involves the use of CTG to seal the extraction socket after tooth extraction, thereby contributing to the maintenance of alveolar bone. However, although this procedure is considered beneficial, it has some disadvantages because of the increased morbidity and the anatomical limitations of the doner sites (Gabay et al. 2022; Meloni et al. 2015; Tal 1999).

A new cross‐linked porcine‐derived collagen matrix (Geistlich Fibro‐Gide [FG], Geistlich Pharma) was introduced to overcome potential complications of autogenous grafts. A preclinical study evaluated the efficacy of this novel material for the augmentation of soft tissues in comparison to CTG (Herford et al. 2019). Histomorphometric analysis of decalcified sections showed that both xenogenic collagen matrix (XCM) and CTG grafts were well integrated into the host tissue and achieved similar levels of tissue volume increase at 2 months after surgery (Thoma et al. 2017). In a clinical study, De Angelis et al. (2021) evaluated the performance of XCM and CTG for soft tissue augmentation in 17 patients per group. They reported that both grafts resulted in similar buccal soft tissue thickness after 1 year of follow‐up. Moreover, they observed that XCM application was associated with decreased amounts of postoperative pain, shorter surgical time, and improved patient satisfaction. However, the validity of these findings is limited by several methodological flaws, such as the lack of randomization and sample size calculation (De Angelis et al. 2021).

This study aimed to investigate the feasibility and efficacy of using a novel cross‐linked collagen matrix (FG) for ARP in comparison with CTG.

## 2 Materials and Methods

### 2.1 Study Design and Sample Size Calculation

This study aimed to compare the clinical and radiographic outcomes of ARP after tooth extraction using CTG, FG, or spontaneous healing (SH) in terms of soft tissue thickness and bone dimensional changes. A randomized clinical trial (RCT) was conducted on 30 patients (25–46 years old) who required extraction of 30 hopeless mandibular posterior teeth (molars and premolars). All teeth were considered beyond repair and had to be extracted. Despite this, the teeth were healthy in terms of periodontal health. The teeth were extracted, and either CTG, FG, or SH was performed according to a random allocation (allocation ratio 1:1:1). The placement of a dental implant took place 6 months after the clinical procedure.

This study was carried out at the Department of Periodontology, Faculty of Dentistry, Damascus University, and was conducted in accordance with the ethical guidelines of the Declaration of Helsinki as revised in 2000. All eligible patients were given the necessary information about the procedure, purpose, and any potential complications of the procedures. In addition, written consent was obtained from all the patients. The protocol of this study was approved by the ethics committee of Damascus University (UDDS‐28066021/SRC‐2486). The present reporting considered the checklist items as proposed in the CONSORT statement (Cuschieri 2019).

The sample size calculation was performed using G*power software (version 3.1.9.7; Germany). A sample size of 21 patients (seven per group) was determined to achieve a Type I error rate of 5% and a power of 80%. The effect size of 1.73 was based on a clinically meaningful 10% expected difference in bone width between the three groups. The highest standard deviation reported of 1.62 mm (Natto et al. 2017; Parashis et al. 2016) was used in the calculation. To account for the roughly 20% probable dropout rate, 30 patients were recruited with 10 patients in each group.

### 2.2 Inclusion and Exclusion Criteria

The study sample included patients who fulfilled the following inclusion criteria:

- The patients had hopeless mandibular posterior teeth (molars or premolars) that required extraction.
- The extraction sockets should have been classified as Type I described by Elian et al. (2007) (facial soft and hard tissues are intact and at normal levels in relation to the cementoenamel junction).
- The patients had a minimum of 2 mm of keratinized tissue at the buccal side of the extraction site.
- The patients had good oral hygiene, scoring 40% or higher on the Chaple and Gispert (2019) index.
- The patients were 18 years of age or older.

The exclusion criteria for the study were as follows:

- The presence of hard tissue defects on the buccal aspect of alveolar bone, such as dehiscence and fenestration, or the loss of the facial bone plate during the tooth extraction.
- Any systematic disease affecting the healing process of the bone.
- Pregnant women.
- Heavy smokers (smoking more than 10 cigarettes a day) (Natto et al. 2017).

### 2.3 Surgical Procedure

#### 2.3.1 Tooth Extraction

**Figure 1:**
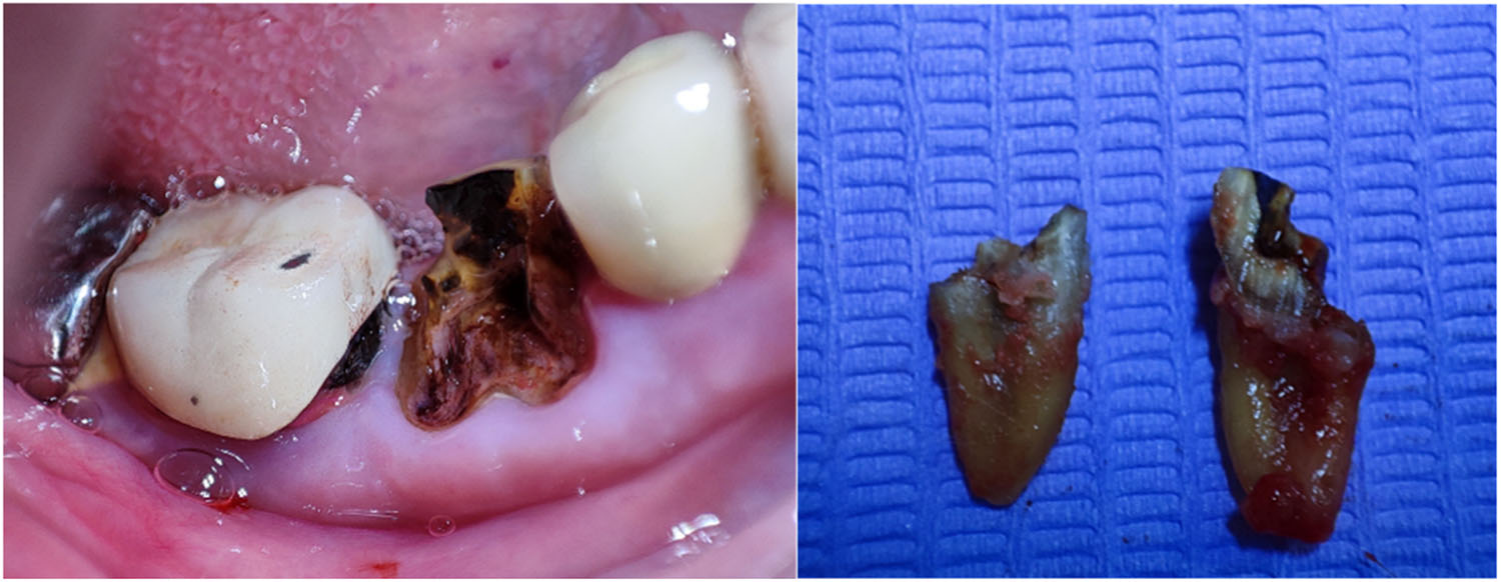
Flapless and atraumatic extraction of the hopeless tooth.

Clinical and radiographic assessments were carried out for all patients before the treatment session. All patients were asked to use povidone iodine mouthwash for 1 min before the procedure. All patients underwent the procedure under local anesthesia using 2% lidocaine, 1:80,000 (Kwang Myung Pharm, Sindaebang 1‐dong Dongjak‐gu, Seoul, Korea). All patients received 2 g of amoxicillin 1 h before the procedure (for patients with penicillin allergy, 600 mg of clindamycin was administered). All extractions were performed in as atraumatic a manner as possible (Figure 1). Following the extraction of teeth, curettage of the sockets was meticulously performed to excise any residual soft tissue, particularly in instances where periapical lesions were detected, in alignment with the recommendations advocating for comprehensive socket debridement and the removal of chronically inflamed tissues (Darby, Chen, and De Poi 2008; Kim and Ku 2020). Then, a saline solution was used to irrigate all extraction sockets. A blinded independent physician was in charge of opening a sealed envelope containing a randomization code to assign the patient to a specific study group: SH, CTG, or cross‐linked collagen matrix FG.

#### 2.3.2 Alveolar Ridge Preservation Procedure

**Figure 2:**
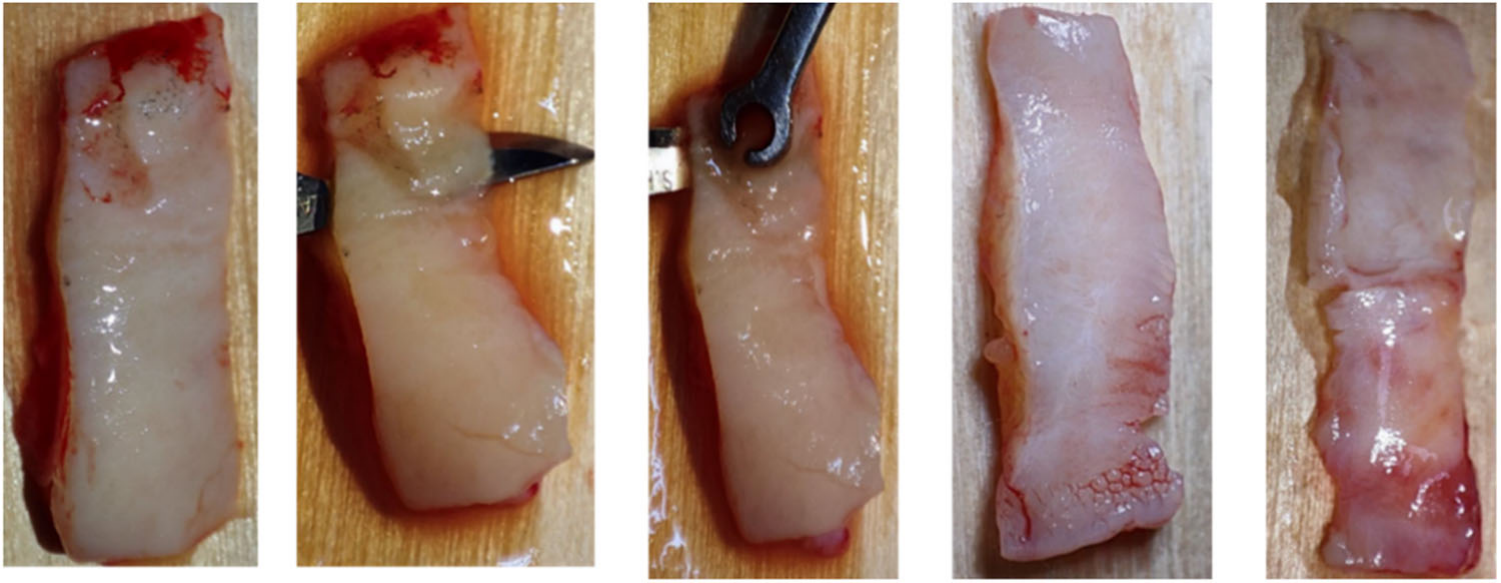
The harvested connective tissue graft de‐epithelization technique.

Extraction sockets were left to heal without any intervention in the SH group. Participants in the second group, referred to as the CTG group, underwent ARP using CTGs devoid of any adjunct SG substances. The application of a barrier material to occlude the socket after extraction, thereby conserving the alveolar bone integrity, was delineated in the investigations conducted by Lekovic et al. (1998) and Raj et al. (2022). The protocol post‐minimally traumatic extraction involved preparation of the recipient site. This commenced with the elevation of a partial‐thickness flap utilizing a 15C surgical blade. The dissection was carefully extended in an apical direction to the mucogingival junction adjacent to the extracted tooth, ensuring the preservation of the interdental papillae and avoiding extension to the adjacent teeth. A CTG was then harvested from the palate according to the technique described by Zucchelli et al. (2010), de‐epithelization was done separately outside of the oral (Figure 2). The graft was fixed in position using a 6/0 Monofilament Polypropylene Nonabsorbable suture material (Vertpro, VertMed GmbH, Germany). The elevated flap was sutured in a more coronal position, a technique used to guarantee comprehensive coverage of the CTG. The procedural strategy implemented in our study has parallels to the technique described by Gamal et al. (2023), wherein a CTG was used for the purpose of ARP. However, it is imperative to note that although our approach is similar to that of Gamal et al. (2023), it is not a direct replication. The distinction lies in the surgical application of the CTG within our methodology, diverging from the parameters set forth by Gamal et al. (2023).

**Figure 3:**
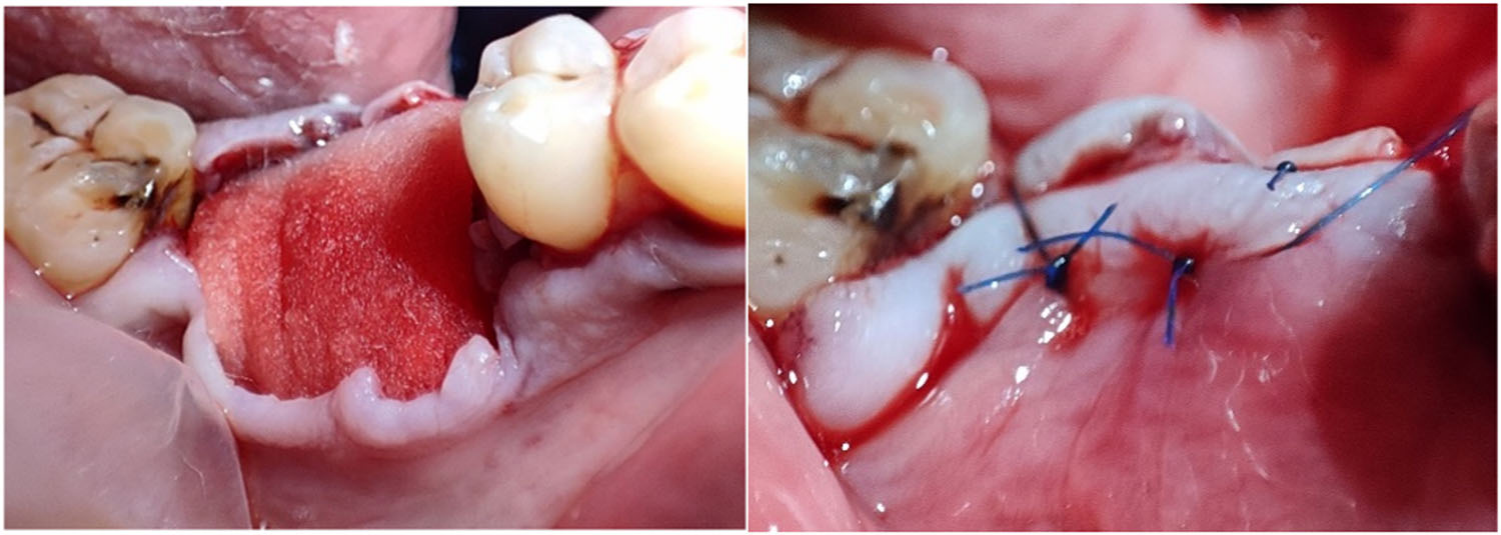
The trimmed Fibro‐Gide matrix fitted in the extraction socket and secured with a 5‐0 absorbable suture material.

In the third group, the extraction socket was covered with an FG collagen matrix that was trimmed to fit the size and shape of the extraction socket. The surgical procedure of the recipient site preparation was the same as that in the CTG group. The FG was secured with a 6/0 Monofilament Polypropylene Nonabsorbable suture material (Vertpro, VertMed GmbH, Germany). The elevated flap was sutured in a more coronal position to achieve wound closure and graft stability, insuring the coverage of the FG collagen matrix (Figure 3). All patients received 500 mg of amoxicillin tid for 8 days. We also instructed the patients to use CHX 0.12% mouthwash twice a day for 3 weeks after the procedure.

**Figure 4:**
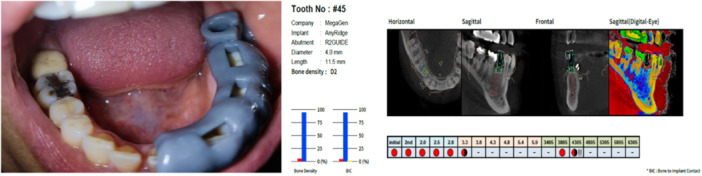
The 3D‐printed surgical guide for implant placement after 6 months.

**Figure 5:**
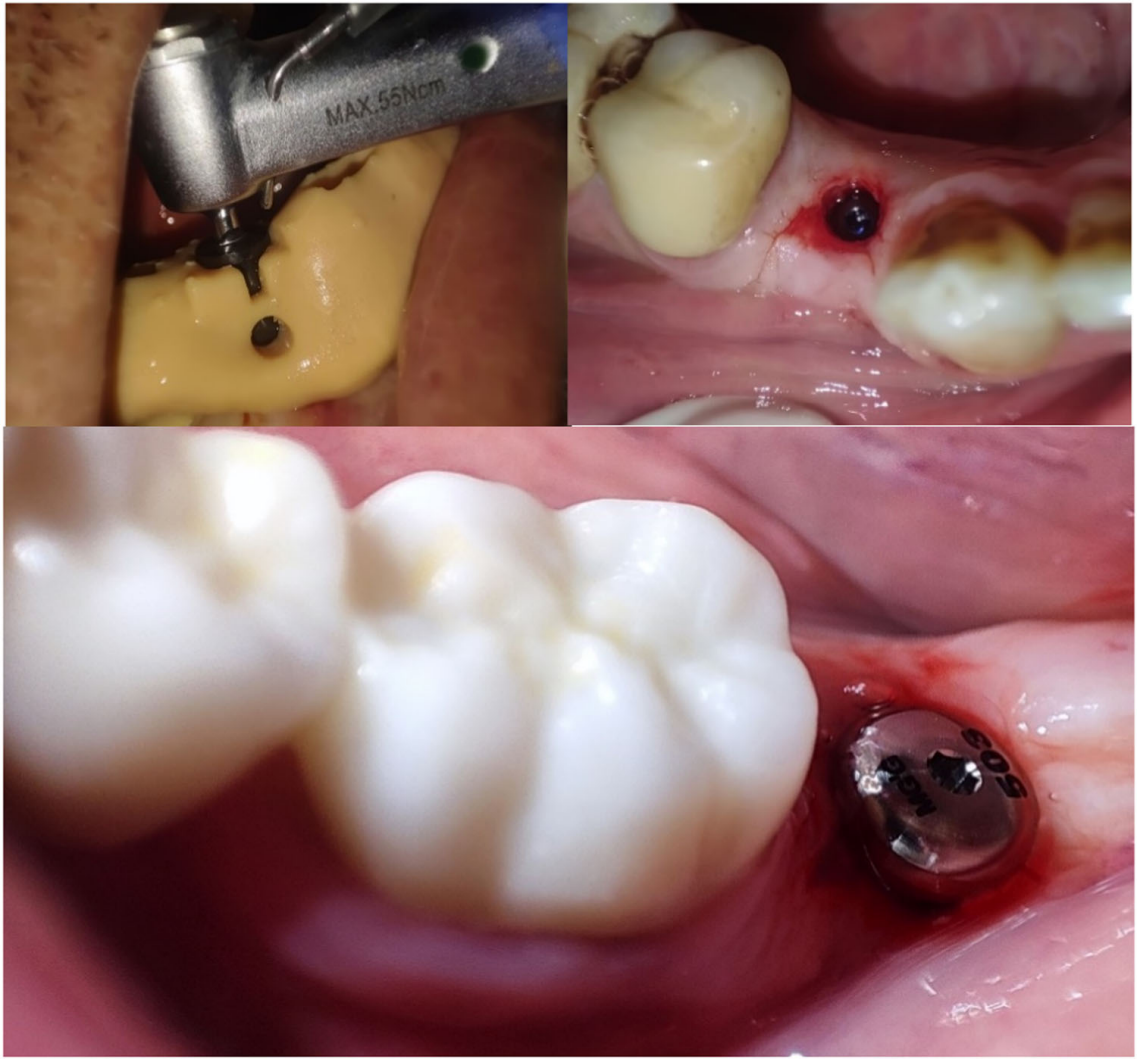
The use of the surgical guide for the dental implant placement and screwing the healing abatement.

Six months after the ARP procedure, AnyRidge implants (Megagen Implant Co., Ltd., Daegu, South Korea) were placed at the location of extracted teeth using a 3D‐printed surgical guide in a flapless approach (Figure 4). The implant site was prepared with a drill sequence following the manufacturer's instructions. A healing abutment was screwed to all implants after placement (Figure 5). The postoperative instructions for the patients were as follows:

- Apply ice packs to the location of the surgery for 20 min every hour for the first 6 h to reduce swelling and pain.
- Rinse the mouth with warm saline solution three times daily starting from the second day after the surgery until the end of the first week to promote wound healing and prevent infection.
- Avoid hard and crunchy foods for the first 48 h after the surgery and consume a soft diet instead to protect the surgical site from trauma.
- Attend follow‐up visits as scheduled to evaluate the wound healing and detect any postoperative problems.

### 2.4 Standardized Clinical and Radiographic Measurements

**Figure 6:**
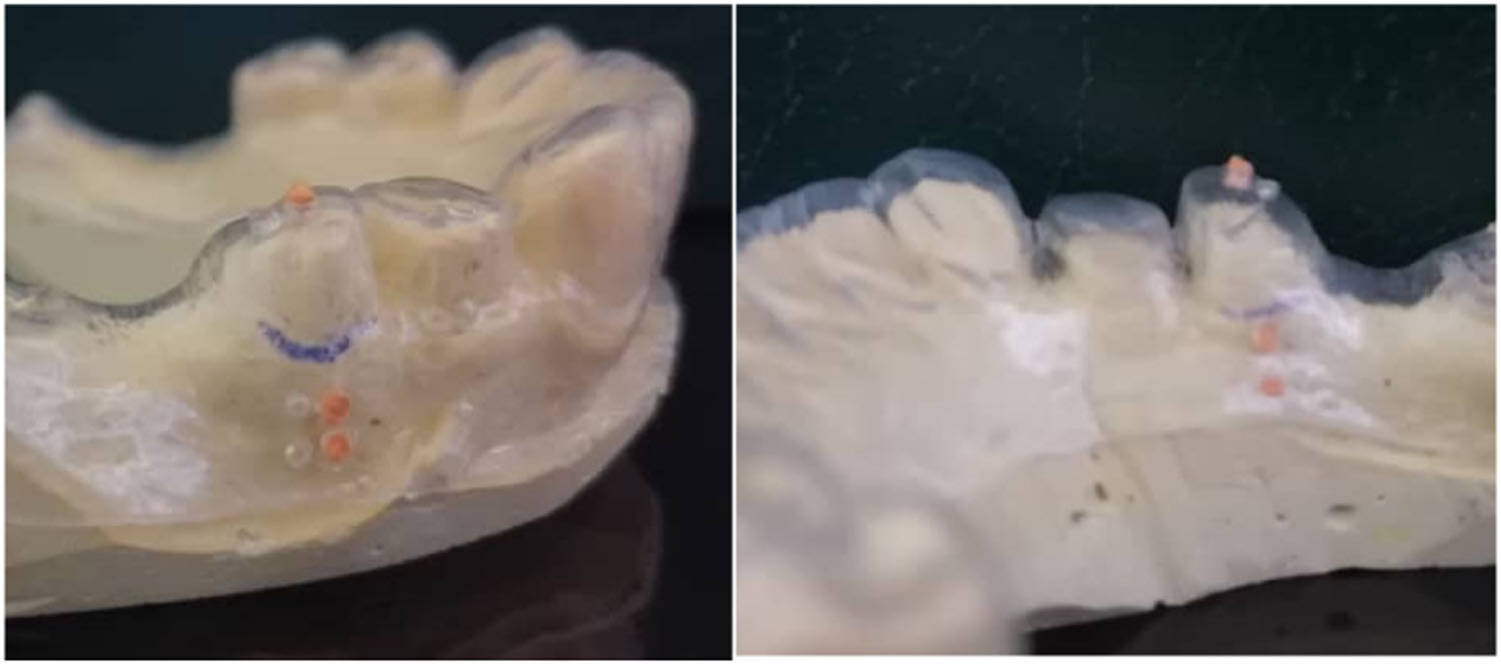
The stent's standardized holes for clinical and radiographic measurements.

The standardization of both clinical and radiographic measurements was achieved using individual stents for each patient; the process of fabricating these stents was reported by Parashis et al. (2016), with a slight modification to fulfill the aims of our study. In brief, the stents for clinical measurements and the cone beam computed tomography (CBCT) scans were made from a plastic shell of 1.5 mm thickness on the patient's cast. The stent had seven standardized holes for consistent clinical and radiographic measurements. Four holes were made on the buccal aspect, corresponding to the tooth to be extracted. The CEJ was set as a reference point in which two of the buccal holes were located 3 mm apical to the reference point, and the remaining two holes were 5 mm apical to the same reference point. One hole of each pair was filled with gutta‐percha points as radiographic landmarks for hard tissue measurements on the CBCT scans and the other hole was empty for measuring soft tissue thickness. Two holes were located on the lingual aspect, corresponding to the same tooth. Gutta‐percha points were used to fill both of these holes to serve as a radiographic landmark on the CBCT scans. The CEJ was used again as a reference point on the lingual aspect, in which one hole was located at 3 and 5 mm apical to the reference point. One hole was located on the occlusal edge, for measuring vertical changes (Figure 6). The customized stents were used to assess the buccal gingival thickness (GT) using endodontic files at the 3 and 5 mm holes. All measurements were repeated twice to the nearest 0.5 mm before and 6 months after the ARP procedure.

Dimensional changes of hard tissues after ARP were quantitatively evaluated utilizing a pair of CBCT scans. The CBCT device used was the PAX‐i3D‐GREEN model from VATECH Co., Ltd., located in Hwaseong, Gyeonggi, South Korea. These scans featured a field of view (FOV) of 15 cm and were conducted with exposure settings of 85 kilovolt peak (kVp) and 15 mA. The initial scan was performed at baseline (preoperative scan—T0), whereas the subsequent scan was executed 6 months following the ARP intervention. Despite the FOV potentially exceeding ARP assessment needs, the research team's access was limited to this specific CBCT device's specifications. This approach was validated in consultation with a faculty radiologist and with informed consent from all subjects.

**Figure 7:**
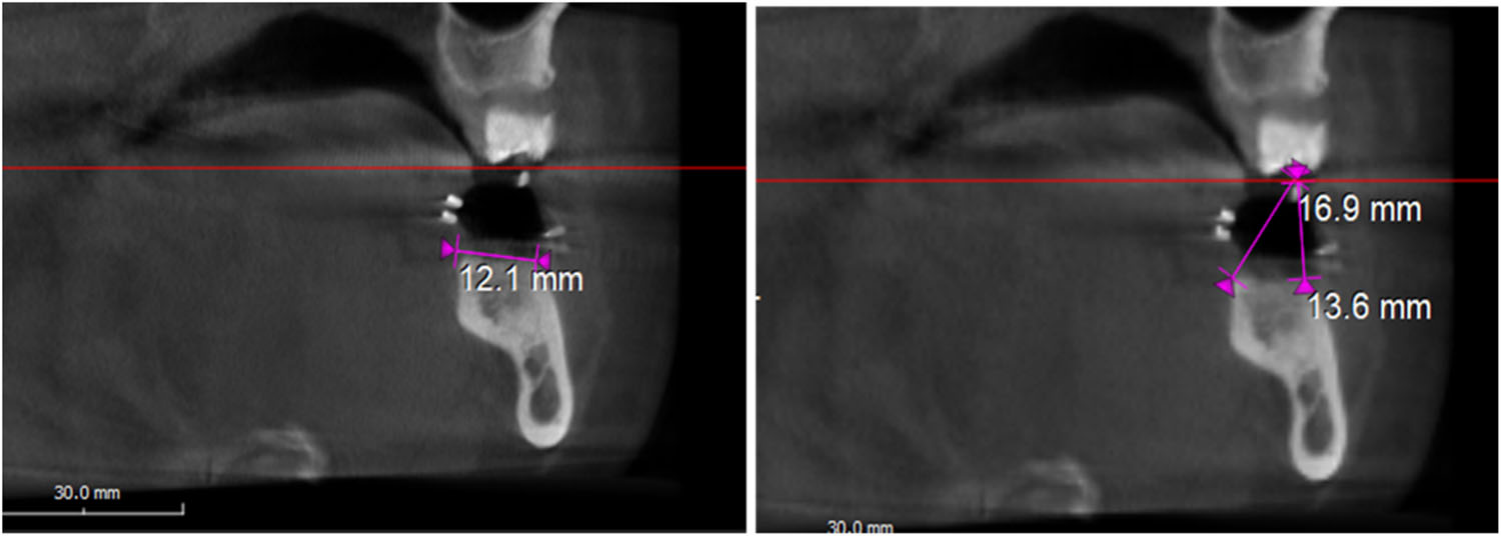
Radiographic measurements of the width of the alveolar ridge, the vertical distance (VR‐BC), and the vertical distance (VR‐LC).

The CBCT scans were performed with the stent in place to standardize the radiographic measurements using the radiographic landmarks. The subsequent parameters were assessed at each time point (Figure 7):

- The width of the alveolar ridge (WB) at the level alveolar crest and at 5 mm apical to the CEJ.
- The vertical distance from the occlusal reference point to the buccal alveolar crest (VR‐BC).
- The vertical distance from the occlusal reference point to the lingual alveolar crest (VR‐LC).

### 2.5 Statistical Analysis

The participants' age was described using mean and range. The treatment procedure was evaluated as a predictor. The measurements of GT at the buccal aspect, horizontal bone width, and vertical bone height were evaluated as outcomes and investigated using inferential statistics. The Kolmogorov–Smirnov test was used to determine data normality. The differences among the three groups (CTG, FG, and SH) in the outcome variables were analyzed using the ANOVA test. Continuous variables were expressed as mean and standard deviation, whereas categorical variables were presented as percentages. The level of significance was set at *p* < 0.05. All statistical analyses were performed with SPSS software (IBM SPSS Statistics 29.0, IBM Corp., Armonk, NY, USA).

## 3 Results

**Figure 8:**
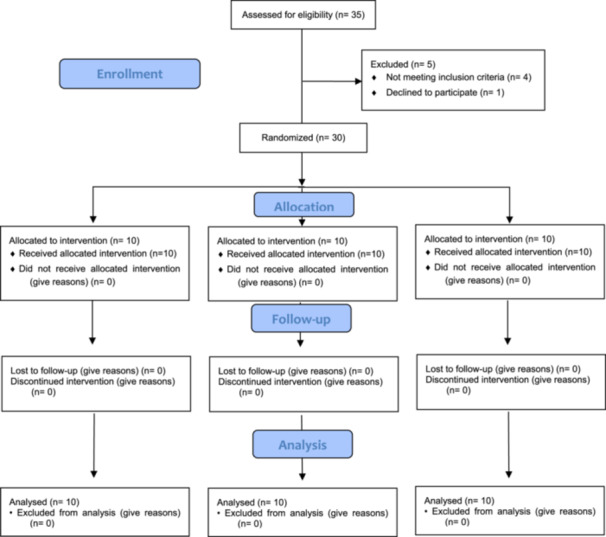
CONSORT 2010 flow diagram.

In this study, 35 participants were screened; of these, five were excluded: three patients smoked more than 10 cigarettes a day, one participant was pregnant, and one participant declined to participate. The remaining 30 participants were randomized into three groups of 10 participants each. The patients of the first two groups underwent a single extraction and a single ARP procedure each. On the other hand, the patients of the third group underwent single extraction each, and the sockets were left to heal spontaneously. All participants completed the study without any complications. Figure 8 shows the participants' enrollment, allocation, and follow‐up in a flow diagram.

### 3.1 Demographic Data

The analysis of demographic data showed comparable age and gender allocation between the three groups. The mean age of the participants was 35.5 years (range 25–46). The age difference among the participants of the CTG group (34.1, range 25–43), the FG group (34.9, range 28–45), and the SH group (35.6, range 26–46) was not significant (*p* = 0.337). The majority of the participants were males (63.3%), with a similar proportion in each group (CTG: 60.0%; FG: 70.0%; SH: 60.0%). The location of the extracted teeth was similar among the groups, with more molars than premolars in each group (CTG: seven molars and three premolars; FG: eight molars and two premolars; SH: six molars and four premolars).

### 3.2 Soft and Hard Tissue Measurements at Baseline

The groups did not differ significantly in GT and both horizontal and vertical bone dimensions before extraction. The SH group had the highest GT at all reference points, whereas the FG group had the lowest horizontal bone width at all reference points. The CTG group had the highest vertical bone height at both buccal and lingual aspects, whereas the FG and SH groups had similar values. The baseline measurements of soft and hard tissues are presented in Table 1.

**Table 1:** Clinical and radiographical measurements at baseline.

Variable (mm), mean ± SD	CTG, *n* = 10	FG, *n* = 10	SH, *n* = 10	*p*
Clinical				
Gingival thickness‐3	1.63 ± 0.77	1.38 ± 0.68	1.75 ± 0.85	0.90
Gingival thickness‐5	1.46 ± 0.72	1.46 ± 0.86	1.81 ± 0.75	0.72
Radiographic				
Bone width‐alveolar crest	8.25 ± 1.22	7.63 ± 1.53	8.29 ± 1.01	0.27
Bone width‐5	8.57 ± 1.91	7.97 ± 1.42	8.63 ± 1.17	0.34
Buccal vertical bone	7.65 ± 1.95	7.17 ± 1.41	7.20 ± 1.12	0.57
Lingual vertical bone	7.95 ± 1.43	7.37 ± 1.24	7.44 ± 1.29	0.42

Abbreviations: ‐3, at the 3 mm apical reference point; ‐5, at the 5 mm apical reference point; CTG, connective tissue graft; FG, Fibro‐Gide; SD, standard deviation; SH, spontaneous healing.

### 3.3 Changes in Soft and Hard Tissue Measurements in 6 Months After ARP

The CTG group showed a significant increase in GT at the 3 and 5 mm reference points, with mean values of 2.42 ± 0.70 mm (*p* = 0.02) and 2.38 ± 0.61 mm (*p* = 0.02), respectively. The FG group also showed a significant increase in GT at the same reference points, with mean values of 3.00 ± 0.71 mm (*p* = 0.00) and 2.88 ± 0.80 mm (*p* = 0.01), respectively. The SH group showed a significant decrease in GT at both reference points, with mean values of 1.31 ± 0.65 mm (*p* = 0.004) and 1.31 ± 0.75 mm (*p* = 0.004), respectively. These results indicated that the SH group had significantly lower GT than the CTG and FG groups at all reference points after 6 months. However, there was no significant difference in GT between the CTG and FG groups. The changes in soft tissue measurements after 6 months are presented in Table 2.

**Table 2:** Clinical soft tissue changes 6 months after the procedure.

Variable (mm), mean ± SD	CTG, *n* = 10		FG, *n* = 10		*p*_1_	SH, *n* = 10		*p*_2_	*p*_3_
	∆	*p*	∆	*p*		∆	*p*		
Gingival thickness‐3	2.42 ± 0.70	0.02*	3.00 ± 0.71	0.00*	0.17	1.31 ± 0.65	0.004*	0.02*	0.01*
Gingival thickness‐5	2.38 ± 0.61	0.02*	2.88 ± 0.80	0.01*	0.27	1.31 ± 0.75	0.004*	0.03*	0.02*

Abbreviations: ‐3, at the 3 mm apical reference point; ‐5, at the 5 mm apical reference point; CTG, connective tissue graft; FG, Fibro‐Gide; *p*_1_, *p* value for changes between CTG and FG; *p*_2_, *p* value for changes between CTG and SH; *p*_3_, *p* value for changes between FG and SH; SD, standard deviation.

* *p* < 0.05.

Table 3 shows the radiographic changes in hard tissue 6 months after the procedure. Bone width reduction was observed in all groups. The mean bone width reduction was higher in the SH group at both reference points compared with the CTG and FG groups (21.00 ± 18.65% vs*.* 24.22 ± 15.05%, 12.26 ± 11.78% vs. 13.43 ± 12.30%, and 12.63 ± 11.87% vs. 13.00 ± 12.61%, respectively), indicating significant differences between the SH group and the other two groups at all reference points after 6 months (*p* = 0.02). However, no significant difference was found between the CTG and FG groups in bone width reduction. The CTG and FG groups had lower mean vertical bone loss at the buccal aspect (11.03 ± 16.41% and 13.59 ± 12.39%, respectively) than the SH group (18.92 ± 24.19%). The CTG and FG groups had lower vertical bone loss on the lingual aspect (12.78 ± 12.56% and 14.62 ± 13.42%, respectively) than the SH group (25.40 ± 30.10%), indicating more bone resorption in the SH group in both dimensions.

**Table 3:** Radiographic changes after 6 months of extraction.

	CTG, *n* = 10			FG, *n* = 10				SH, *n* = 10				
Variable, mean ± SD	∆ (*mm*)	∆ (%)	*p*	∆ (*mm*)	∆ (%)	*p*	*p*_1_	∆ (*mm*)	∆ (%)	*p*	*p*_2_	*p*_3_
Bone width‐alveolar crest	+0.86 ± 2.31	12.26 ± 11.78	0.01*	+0.93 ± 2.38	12.63 ± 11.87	0.01*	0.32	+1.53 ± 2.29	21.00 ± 18.65	0.01*	0.02*	0.02*
Bone width‐5	+1.09 ± 1.88	13.43 ± 12.30	0.01*	+1.01 ± 1.72	13.00 ± 12.61	0.01*	0.67	+1.96 ± 2.45	24.22 ± 15.05	0.01*	0.02*	0.02*
Buccal vertical bone	−0.30 ± 1.09	11.03 ± 16.41	0.01*	−0.47 ± 2.30	13.59 ± 12.39	0.01*	0.5	−1.46 ± 1.67	18.92 ± 24.19	0.01*	0.02*	0.02*
Lingual vertical bone	−0.79 ± 3.07	12.78 ± 12.56	0.01*	−0.96 ± 0.97	14.62 ± 13.42	0.01*	0.16	−2.77 ± 3.17	25.40 ± 30.10	0.01*	0.02*	0.02*

Abbreviations: ‐5, at the 5 mm apical reference point; ‐alveolar crest, at the alveolar crest reference point; CTG, connective tissue graft; FG, collagen sponge; *p*_1_, *p* value for changes between CTG and FG; *p*_2_, *p* value for changes between CTG and control; *p*_3_, *p* value for changes between FG and control; SD, standard deviation.

* *p* < 0.05.

## 4 Discussion

This study aimed to investigate the feasibility and efficacy of using a novel cross‐linked collagen matrix (FG) for ARP compared to the use of CTG. To the best of our knowledge, our study is the first randomized controlled trial to evaluate this novel biomaterial for this indication; we could not find any other published papers comparing the utilization of FG and CTG in APR, which limits our ability to compare the present results with other similar studies. Our findings highlight the minimized alveolar ridge resorption after the application of ARP using CTG or FG in comparison to SH after tooth extraction.

FG is a biomaterial derived from porcine dermis that can be utilized as a scaffold for the regeneration of soft tissues. It consists of a porous matrix of type one and three collagen, which mimics the architecture of human connective tissue. The collagen fibers are chemically cross‐linked to enhance the stability and volume retention of the matrix. The surface morphology of FG reveals a rough and open‐porous structure that facilitates the infiltration of blood vessels and cells into the scaffold (dos Santos et al. 2023). The selection of CTG or XCM may be a multifactorial decision, including tissue biotype, the size of the defect, the experience of the clinician, and the preference of the patient. The use of XCM could offer a replacement for the use of CTG by minimizing the morbidity of the patient and reducing the time consumed during the operation (Ashurko et al. 2023). Although the study by Ashurko et al. does not explicitly address ARP techniques, it provides a valuable comparative analysis of the clinical efficacy of CTG and XCM, thereby contributing to the broader discourse on periodontal biomaterials.

The present results revealed the significant effect of the studied procedure on the buccal GT and the hard tissue dimensions after 6 months. The SH group had the lowest GT and the highest bone resorption in both horizontal and vertical directions, indicating that SH resulted in poor outcomes regarding the soft tissue and bone stability. The CTG and FG groups had similar GT and bone width reduction, suggesting that both the options were effective in preserving the ridge width. However, the CTG group had lower vertical bone loss than the FG group, implying that the CTG provided better protection for the underlying bone. These findings are consistent with previous studies that reported the advantages of using CTGs over other materials for ridge preservation (Eghbali et al. 2018). However, the FG group showed acceptable outcomes, especially in terms of GT, which is an important factor regarding the aesthetic outcomes of dental implants.

The observed vertical bone resorption on the buccal aspect in our study aligns with the findings of antecedent research, which assessed a variety of surgical approaches and biomaterials pertinent to ARP, corroborating the results reported in subsequent studies. The surgical approaches included flap or flapless surgery and primary or secondary wound closure. The materials included barrier membranes and/or bone grafts (Araújo and Lindhe 2009; Artzi, Tal, and Dayan 2000; Barboza et al. 2010; Barone et al. 2013, 2014; Brownfield and Weltman 2012; Darby, Chen, and De Poi 2008; Engler‐Hamm et al. 2011; Fickl et al. 2008; Iasella et al. 2003; Mardas, Chadha, and Donos 2010; Weng, Stock, and Schliephake 2011). ARP procedures have been shown to reduce, but not eliminate, the dimensional bone changes following the extraction of single‐ and multi‐rooted teeth compared to spontaneous socket healing. However, total preservation of the alveolar ridge dimensions after the extraction of a tooth remains a challenge even when ARP procedures are utilized (Hämmerle, Araújo, and Simion 2012). This study corroborates this finding and suggests that further improvements are needed to achieve optimal outcomes. This study reported similar hard tissue dimensional changes to those of recent studies that investigated the effect of ARP using different soft tissue closure techniques after tooth extraction. These techniques involved either an autogenous free gingival graft (6–8 mm in diameter) or an autogenous soft tissue punch graft modification (similar to the collagen matrix) to cover the extraction socket (Schneider et al. 2014).

All the procedures in our study, including the surgical phase and the clinical measurements were performed by the same investigator. This could have introduced a potential bias because of the lack of blinding. However, this could also reduce the variability among the measurements. Moreover, the use of individual stents for soft tissue and hard tissue measurements at baseline and 6 months after the surgical procedure minimized the sources of error in the clinical and radiographic assessments. Furthermore, a blinded investigator measured the hard tissue changes on CBCTs.

FG is a synthetic collagen matrix that mimics the natural extracellular matrix and promotes soft tissue regeneration (Vallecillo et al. 2021). Therefore, this material could be a viable alternative to CTGs, especially for patients who prefer to not go through the process of autogenous soft tissue graft harvesting or have limited donor tissue availability. Further studies with a larger patient sample size are needed to validate the long‐term efficacy of the FG for ridge preservation.

## 5 Conclusion

In light of the limitation discussed above, we conclude that the use of FG compared to the CTG for ARP can achieve similar results in terms of the thickness of soft tissue. Moreover, there is no difference in the dimensional bone changes in the horizontal aspect between the two options.

## Author Contributions

Ammar Ibrahim conceived the research idea, performed the treatment, and interpreted the data and drafted, revised, formatted, and edited the manuscript. Rowaida Saymeh co‐conceived the research idea and supervised Ammar Ibrahim's PhD thesis. Both authors gave their approval to the final version of this manuscript.

## Ethics Statement

This study was approved by the Institutional Ethics Committee of Damascus University (Approval Number UDDS‐28066021/SRC‐2486).

## Consent

All eligible patients were given the necessary information about the procedure, purpose, and any potential complications of the procedure procedures. In addition, written consent was obtained from all the patients.

## Conflicts of Interest

The authors declare no conflicts of interest.

## Data Availability

Pseudonymized data can be obtained upon reasonable request from the corresponding author.
